# Associations between program outcomes and adherence to Social Cognitive Theory tasks: process evaluation of the SHED-IT community weight loss trial for men

**DOI:** 10.1186/s12966-014-0089-9

**Published:** 2014-07-11

**Authors:** Philip J Morgan, Hayley A Scott, Myles D Young, Ronald C Plotnikoff, Clare E Collins, Robin Callister

**Affiliations:** 1 Priority Research Centre in Physical Activity and Nutrition, The University of Newcastle, Callaghan 2308, NSW, Australia; 2 School of Education, Faculty of Education and Arts, The University of Newcastle, Callaghan 2308, NSW, Australia; 3 Nutrition and Dietetics, School of Health Sciences, Faculty of Health and Medicine, The University of Newcastle, Callaghan 2308, NSW, Australia; 4 School of Biomedical Sciences and Pharmacy, Faculty of Health and Medicine, The University of Newcastle, Callaghan 2308, NSW, Australia

**Keywords:** Social cognitive theory, Men, Obesity, Online, Process evaluation, Weight loss

## Abstract

**Background:**

Despite rising international rates of obesity, men remain reluctant to participate in weight loss research. There is a lack of evidence to guide the development of effective weight loss interventions that engage men. The objective of this study was to provide a comprehensive process evaluation of the SHED-IT (Self-Help, Exercise and Diet using Information Technology) weight loss program for men, as delivered in the SHED-IT community weight loss trial, and to identify key components associated with success.

**Methods:**

In an assessor-blinded randomised controlled trial, 159 overweight and obese men (BMI 25.0-40.0 kg/m^2^) were randomised to one of two gender-tailored weight loss interventions with no face-to-face contact, or a control group. The interventions were informed by Bandura’s Social Cognitive Theory (SCT) with men encouraged to complete a Support Book containing SCT-based tasks including goal setting, reward setting, creation of social support strategies and self-monitoring of: i) weight, ii) physical activity, and iii) diet. At post-test, compliance with SCT tasks was examined and men also completed a process evaluation questionnaire.

**Results:**

Both SHED-IT intervention groups demonstrated greater weight loss during the intervention compared to the control, with no difference between intervention groups. Most men engaged with the SCT tasks although compliance declined over time and utilisation of social support networks and reward selection was poor. In a multiple regression model, the number of goals set (β [95% CI] = -0.3 [-0.6, -0.1], p = 0.01) and the number of weight records documented (β [95% CI] = -0.2 [-0.4, -0.0], p = 0.03) independently predicted weight loss. The process evaluation indicated that men found the programs to be supportive, enjoyable and beneficial.

**Conclusions:**

This process evaluation provides valuable information to inform the development of obesity treatment strategies that engage men. Future studies with men should include a strong focus on self-monitoring and goal setting to enhance behaviour change and improve treatment effects.

**Trial registration:**

Australian New Zealand Clinical Trials Registry ACTRN12610000699066.

## Background

Obesity is an increasingly prevalent global health problem [[1]]. Obesity more than triples the risk of developing type 2 diabetes, gall bladder disease and sleep apnoea, and doubles the risk of heart disease, hypertension and asthma [[2],[3]]. This is particularly concerning for males who exhibit a visceral abdominal pattern of obesity, which further increases the risk of developing obesity-related chronic diseases [[2]]. Despite the need for weight management, men are less likely to perceive themselves as overweight [[4]] or to participate in weight loss interventions or research [[5]]. This may arise from a lack of appeal of traditional weight loss programs and/or reduced motivation after previous failed weight loss attempts [[6]]. Previous studies indicate that face-to-face weight loss programs, particularly group-based programs, do not appeal to men [[5]-[8]]. Consequently, there is a need to develop weight loss programs targeted to men’s physical and psychological profile [[9],[10]]. Men also report preferences for convenient programs that include personalised feedback, people they can relate to (particularly other men), the use of humour and minimal disruption to their current lifestyle [[6],[8],[11]].

Currently, there is a lack of evidence to guide the development of effective weight loss interventions for men. Recent systematic reviews have clearly demonstrated that men are consistently under-represented in weight loss research [[5],[9]]. As such, many of the current recommendations provided to men attempting weight loss are based on trials that were predominantly conducted with women. A recent systematic review of male-only weight loss studies documented the success of gender-targeted approaches, but noted more high-quality studies are required to improve understanding of how to successfully engage men with effective weight loss programs [[9]]. Process evaluation of such studies is also an integral component of intervention research as it establishes the quality of the intervention and informs the design and implementation of future trials [[12]]. Process evaluation may examine participants’ views of the program, whether the program was implemented as intended and the effectiveness of individual program components in relation to study outcomes [[12]]. As there is limited evidence regarding effective weight loss interventions for men [[5],[9]], process evaluation of male-only trials is essential to determine (i) which treatment modalities are feasible and acceptable to men, and (ii) which intervention components are associated with successful treatment outcomes in men.

We developed a male-only weight loss program called the SHED-IT (Self-Help, Exercise, and Diet using Information Technology) Program that involved no face-to-face contact and was tailored for men. This program was successfully tested in a pilot study with University staff and students [[10],[11],[13]-[15]] and was subsequently refined and tested in an effectiveness trial with a large community sample of overweight and obese men [[16],[17]]. Although both trials tested the SHED-IT program, important intervention changes were made between trials. For example, for the current trial, the face-to-face session was removed from the intervention and replaced with a DVD to improve scalability. In addition, Bandura’s Social Cognitive Theory (SCT) was more rigorously operationalised in the current intervention, with participants completing a newly developed weight loss Support Book containing SCT tasks. The study protocol [[16]] and outcomes [[17]] of the current trial have been reported elsewhere. The SHED-IT program targeted behaviour change mediators outlined in Bandura’s SCT [[18]], which provides a framework for changing an individual’s cognitions in order to improve adherence with optional behaviours. Men must value the outcome (i.e. weight loss) if they are to make changes to dietary and exercise behaviours. They must believe weight loss will occur by making these changes and that they have the confidence, knowledge and skills required to implement these changes.

In the current study, we present the findings of a comprehensive process evaluation 3-months post-intervention of the SHED-IT community weight loss randomised controlled trial (RCT) [[16]]. Specifically, we investigated the following three research aims:

1) To report participant engagement with SCT-based program tasks including goal setting, reward setting, social support and self-monitoring of: i) weight, ii) physical activity, and iii) dietary intake;

2) To examine the association between compliance with SCT-based tasks and weight loss;

3) To examine men’s perceptions of the quality of the SHED-IT program and its impact on their weight as well as their knowledge and skills about weight loss.

## Methods

### Study design

The SHED-IT community trial has been described in detail previously [[16],[17]]. Briefly, this assessor-blinded RCT included a community sample of overweight and obese men (BMI 25.0-40.0 kg/m^2^) from the Hunter region of New South Wales, Australia. Participants were randomly allocated to one of three study arms: i) SHED-IT *Resources*; ii) SHED-IT *Online*, or iii) a wait-list control group. This study was approved by the Human Research Ethics Committee, University of Newcastle and all subjects provided written informed consent. The trial was registered with the Australian New Zealand Clinical Trials Registry, Number ACTRN12610000699066.

### Intervention descriptions

The SHED-IT interventions were based on SCT, in which Bandura proposes that behaviour change is influenced by attributes of the behaviour itself, personal factors and environmental factors [[18]]. In order to target the key behaviour change constructs outlined in SCT, the SHED-IT interventions first aimed to educate participants about the physical, social, and self-evaluative (i.e. personal) benefits of weight loss through increased physical activity and healthy eating. In addition, the resources were designed to increase participants’ self-efficacy for performing these behaviours and assist them to develop the self-regulatory skills needed to sustain the behaviours over time and in the face of potential barriers. These self-regulatory behaviours include goal setting, self-monitoring and reward provision [[19]]. Finally, given the importance of socio-structural facilitators in establishing long-term behaviour change, the SHED-IT resources were designed to help men engage with their social networks and establish helpful social support strategies [[18]]. Both the *Resources* and *Online* SHED-IT interventions included the same core components and were based on the same theoretical constructs. Men were instructed to complete all program tasks for 3-months which, excluding data collection visits, were completed without face-to-face contact.

### SHED-IT Resources intervention

Men randomised to the *Resources* group received a resource package containing the *‘SHED-IT Weight Loss Handbook for Blokes’*, a 25-minute *‘Weight Loss DVD for Blokes’*, a kilojoule counter book, a pedometer and a tape measure. These resources focused on nine key weight loss messages: *1) Read food labels, 2) Keep a healthy lifestyle diary, 3) Reduce kilojoule-dense snacks, 4) Be prepared, 5) Every step counts, 6) Reduce your sitting time, 7) Surf the urge* (in reference to resisting the urge to snack unnecessarily), *8) Reduce your portion sizes,* and *9) Don’t drink your kilojoules*.

In addition to these resources, men also received the “*SHED-IT Weight Loss Support Book for Blokes*”, henceforth referred to as the Support Book, which included the following six sections:

i) *Calculating kilojoule output*

ii) *Weight and waist record charts*: Participants updated charts weekly.

iii) *Pedometer diary*: Participants monitored step counts four days each week, calculated the average, and plotted the weekly average on a chart.

iv) *Goal setting:* Participants were advised to set three goals monthly, one for weight loss, one for physical activity and one for dietary intake. Instructions for setting SMART goals (i.e. Specific, Measureable, Attractive, Realistic and Time-targeted) and examples were provided. Men were also instructed to mark off each achieved goal and record a reward they would receive at the end of each month if they successfully achieved all three goals.

v) *Social support strategies*: Men were instructed to record one or two social support strategies each month. Instructions and examples were provided.

vi) *Healthy lifestyle diary:* Instructions were provided for recording dietary intake and exercise participation on four days of each week for the 3-month intervention. This was paper-based and included space to calculate whether the daily kilojoule target for weight loss was achieved.

### SHED-IT Online intervention

Those randomised to the *Online* group received a website user guide in addition to the resource package described above. The user guide included information on using the freely available Calorie King™ (Australia) website (http://www.calorieking.com.au) and instructed participants to record dietary intake and exercise online for four days per week, in place of the paper-based diary completed by the *Resources* group. Participants also had the option of self-monitoring their weight, waist and pedometer steps on the website rather than the Support Book, if they chose. Contingent on diary completion, research assistants provided men with seven individualised feedback emails based on their online food and exercise diary entries. These were provided weekly during the first month, fortnightly during the second month and once during the third month.

### Data collection

Men were measured at baseline, 3 months (immediate post-intervention) and 6 months (3-month follow-up). All measurements were performed by trained research assistants who were blinded to group allocation.

### Measurement of anthropometrics, demographics and behaviours

Body weight (CH-150kp, A&D Mercury Pty Ltd, Australia) and height were measured, and Body Mass Index (BMI) was calculated. Waist circumference was measured at the umbilicus and the largest circumference between the umbilicus and lower costal border [[20]] (KDSF10-02, KDS Corporation, Osaka, Japan), and body fat (kg) was determined by bio-impedance analysis (InBody720, Biospace Co., Ltd, Seoul, Korea) [[21]]. Age and socioeconomic status [[22]] were determined by questionnaire. Dietary intake was measured using the validated Australian Eating Survey [[23]], while physical activity levels were objectively measured via seven days of pedometry with Yamax digiwalker SW200 pedometers (Yamax Digi-Walker SW200, Kunamoto City, Japan).

### Program compliance measures

Compliance with SCT-based tasks was quantified by examining adherence to each of the six Support Book components during the 3-month intervention phase. Specifically, this comprised self-monitoring of weight, waist circumference and pedometer step counts; setting of goals, rewards, and social support strategies; and monitoring dietary intake and exercise participation. Compliance with each Support Book component was defined as recording each component at least 80% of recommended reporting times. For example, compliance with monitoring waist circumference was defined as weekly recording of waist circumference measurements at least 11 times over the 3-month (13-week) period. This cut-off was chosen based on data from the SHED-IT pilot study [[13]], where men who completed 80% of weight entries and 80% of the food and exercise diary recommendations demonstrated significantly greater program outcomes compared to those that did not. Participants were advised to set SMART goals, with examples provided, such as “*this month, I will go for a half-hour walk around the neighbourhood, three times per week*”. Quality social support strategies were defined as engaging a person from one’s social support network to participate in a supportive activity that would aid in successful completion of the SHED-IT program requirements.

A project-specific process evaluation questionnaire, adapted from an instrument used in previous research [[11]], was completed at the 6-month assessments. This questionnaire measured perceptions of the SHED-IT program in general; satisfaction with the program resources including the DVD, the Handbook and the Support Book, and the effect of the program on the people around them. The *Online* group completed additional questions regarding perceptions of the Calorie King website and personalised feedback reports.

### Statistical analysis

Data were analysed using Statistical Package for the Social Sciences (SPSS version 20.0 software; SPSS, Chicago, IL, USA) and are reported as mean (standard deviation), median (interquartile range) or the percentage of participants for each specified variable. For continuous variables, groups were compared using analysis of variance (ANOVA) with Bonferroni post-hoc testing. Proportions of subjects recording each Support Book component were examined using Pearson’s chi-squared test (Aim 1). The correlation between change in selected outcomes (e.g. weight, waist circumference, physical activity) and engagement with each Support Book component (i.e. goal setting and rewards, social support, monitoring of weight and pedometer steps, dietary intake and exercise participation) was assessed using the Spearman rank test (r_s_) (Aim 2). Backward stepwise regression was performed using Stata (Intercolled Stata version 11.2, Stata Corporation, College Station, TX, USA) to determine the independent effect completing each Support Book component had on weight change (kg) (Aim 2). These analyses were adjusted for age and socio-economic status (SES), which are known to interact with weight [[24]] and treatment outcomes in previous weight loss trials with men [[25],[26]]. Intervention group was also adjusted for in the analyses. In addition, the robust variance estimator was applied to account for the data being skewed. As records for waist circumference measurement and exercise participation were highly correlated with records for weight measurement and dietary intake respectively, they were not included in the regression model. Process evaluation questions were analysed by independent samples t-tests to determine the main effect of intervention allocation (i.e. *Online* vs. *Resources*) on participants’ perceptions of the SHED-IT program (Aim 3). *P*-values <0.05 were considered statistically significant for all analyses (Aim 3).

## Results

### Participant characteristics

The high appeal of a male-only weight loss program of this nature was demonstrated by the recruitment of 159 men from more than 600 enquiries within a one-week period. A total of 53 men were randomised to the *Online* group, 10 of which withdrew [[17]] and 11 did not return their Support Book, leaving 32 included in the analysis of program compliance. For the *Resources* group, 54 men were randomised, 11 withdrew [[17]], and eight did not return their Support Book, totalling 35 men included in the compliance analysis. For those included in the analysis of compliance, more men of a high SES completed the *Online* program and returned their Support Book compared to the Resources program. However as mentioned previously, SES was adjusted for in the analyses, where significant.

At baseline, these participants (n = 67) had a mean (SD) age of 47.3 (10.9) years, BMI of 32.6 (13.5) kg/m^2^, walked 6950 (2962) steps per day and consumed 11547 (3496) kJ per day. The only significant difference between groups at baseline was SES, with more participants in the *Resources* group living in higher socio-economic areas than men in the *Online* group. Additional information on participants’ baseline characteristics can be found in Additional file 1: Table S1.

As previously reported, after 6 months, weight was significantly reduced (p < 0.001) in both the *Online* group (-4.7 kg, 95% CI -6.1, -3.2) and *Resources* group (-3.7 kg, 95% CI -4.9, -2.5) relative to the control group (-0.5 kg, 95% CI -1.4, 0.4), with no significant difference between intervention groups (*p* > 0.05) [[17]]. There was also a significant decrease in energy intake in both the *Online* (-1673 kJ/day, 95% CI -2268, -1078) and *Resources* (-1190 kJ/day, 95% CI -1924, -456) groups. In addition, significant increases in physical activity were observed for the *Online* group (1575 steps/day, 95% CI 753, 2397) and *Resources* group (1586 steps/day, 95% CI (796, 2376). As with weight, the differences between interventions were not significant for these outcomes (*p* > 0.05 for both) [[17]].

### Engagement with SCT-based program tasks

Table 1 shows the *Online* and *Resources* groups had similar levels of compliance with recording goals and social support strategies, and monitoring weight, waist circumference and dietary intake. Approximately half of the men set weight, exercise and nutrition-related goals during the 3-month program, with more than half of these reporting having achieved these goals. Very few participants recorded social support strategies, with compliance decreasing from month one (44%) to month three (25%). The proportion of men who were compliant with monitoring their weight (63% vs. 50%, *p* = 0.31), waist circumference (56% vs. 43%, *p* = 0.27), pedometer steps (45% vs. 49%, *p* = 0.78) and dietary intake (63% vs. 50%, *p* = 0.31) was similar for the *Online* and *Resources* groups. However, the *Online* group were more compliant with monitoring their exercise participation than the *Resources* group (41% vs. 18%, p = 0.046).

**Table 1:** Engagement with program components using the Support Book

	**Online (n = 32)**	**Resources (n = 35)**	***P*-value**^**e**^
**Goal Setting (%)**^**a**^			
***Month 1***			
Weight Goal	59.4	61.8	0.84
*Achieved*^***b***^	63.1	66.7	0.76
PA Goal	56.3	61.8	0.65
*Achieved*^***b***^	72.1	71.4	0.77
Nutrition Goal	53.1	61.8	0.48
*Achieved*^***b***^	76.5	71.4	0.77
Reward	25.0	35.3	0.36
***Month 2***			
Weight Goal	53.1	44.1	0.46
*Achieved*^***b***^	76.5	66.7	0.34
PA Goal	50.0	41.2	0.47
*Achieved*^***b***^	81.2	78.6	0.49
Nutrition Goal	50.0	41.2	0.47
*Achieved*^***b***^	81.2	78.6	0.49
Reward	25.0	23.5	0.89
***Month 3***			
Weight Goal	50.0	41.2	0.47
*Achieved*^***b***^	43.8	64.3	0.66
PA Goal	46.9	41.2	0.64
*Achieved*^***b***^	59.9	57.0	0.67
Nutrition Goal	43.8	38.2	0.65
*Achieved*^***b***^	57.1	61.5	0.89
Reward	15.6	17.6	0.83
**Social Support (%)**			
***Month 1***			
Two Strategies Recorded	37.5	31.4	0.71
One Strategy Recorded	6.3	11.4	
No Strategies Recorded	56.3	56.7	
***Month 2***			
Two Strategies Recorded	25.0	20.0	0.88
One Strategy Recorded	3.1	2.9	
No Strategies Recorded	71.9	77.1	
***Month 3***			
Two Strategies Recorded	18.8	14.3	0.27
One Strategy Recorded	6.3	0	
No Strategies Recorded	75.0	85.7	
**Calculating kJ Output (%)**	77.4	82.9	0.58
**Self-Monitoring**^**c**^			
Weight	11 (3, 13)	10 (4, 13)	1.00
Waist Circumference	7 (1, 12)	10 (1, 12)	0.89
Pedometer Steps	6 (0, 12)	10 (0, 12)	0.66
**Compliance With Self-Monitoring**^**d**^			
Weight	62.5	50.0	0.31
Waist Circumference	56.3	42.9	0.27
Pedometer Steps	45.2	48.6	0.78
Dietary Intake	63.0	50.0	0.31
Exercise Participation	40.7	17.6	**0.046**

^a^Recorded as % the percentage of participants completing the specified Support Book component.

^b^Reported as the percentage of participants achieving the specified goal.

^c^Median (IQR) number of weekly records made during the 3-month study (out of a possible 13).

^d^Proportion of participants regarded as compliant with self-monitoring. Compliance was defined as completing the specified Support Book component ≥80% of the time.

^e^Bolded *p* value is statistically significant (*p <0.05)*.

As there were essentially no differences between intervention groups for program engagement, or outcomes achieved [[17]], data were pooled for clarity. Table 2 shows that 51% of all weight loss goals set aimed for the recommended 0.5-1.0 kg weight loss per week, although 24% of goals aimed for >1 kg of weight loss per week. Approximately 60% of all exercise goals aimed to increase walking or reach a predetermined pedometer step target, while bike riding and strength training were also popular. The proportion of SMART exercise goals decreased significantly during the third month of the program, but on average 80% of all goals were SMART. Nutrition goals were recorded approximately half of the time. However, only 56% of these goals were SMART and decreased significantly throughout the program. The most popular nutrition goals related to reducing intake of ‘junk food’ (e.g. chips, pies, soft drink) and increasing intake of fruits and vegetables. Documenting rewards for goal achievement was low, with less than one-third of men recording goal achievement in any given month. Approximately half of rewards listed related to a night out for dinner or alcohol.

**Table 2:** Goals and rewards recorded during the SHED-IT program (n = 66)

	**Overall**	**Month 1**	**Month 2**	**Month 3**	***P*-value**^**c**^
**Weight Goals**					
Recorded	51.5	60.6	48.5	45.5	0.18
What?					
*<0.5 kg/week*	20.6	15.0	21.9	26.7	0.30
*0.5-1 kg/week*	51.0	52.5	53.1	46.7	
*>1 kg/week*	23.5	27.5	12.5	10.0	
*Other*	4.9	0	6.3	10.0	
SMART^a^	96.1	100.0	93.8	93.2	0.08
**Exercise Goals**					
Recorded	49.5	59.1	45.5	43.9	0.16
What?					
*Pedometer Count*	31.9	31.0	40.0	25.6	0.26
*Walk*	25.9	33.3	20.0	23.1	
*Bike Ride*	13.8	14.3	11.4	15.4	
*Gymnasium/Strength Training*	13.8	7.1	14.3	20.5	
*Run/Jog*	2.6	0	0	7.7	
*Other*	12.1	14.3	14.3	7.7	
SMART^a^	79.6	84.6	83.3	65.6	**0.03**
**Nutrition Goals**					
Recorded	48.0	57.6	45.5	40.9	0.14
What?					
*Junk Food*^b^	18.7	32.0	5.7	12.5	
*Fruit/Vegetables*	11.1	6.0	17.1	12.5	0.15
*Kilojoule Target*	9.4	8.0	8.6	12.5	
*Water*	8.5	12.0	2.9	9.4	
*Portion Size*	8.5	6.0	8.6	12.5	
*Alcohol*	7.7	4.0	11.4	9.4	
*Caffeine*	6.8	8.0	8.6	3.1	
*Breakfast*	5.1	8.0	5.7	0	
*Other*	23.9	16.0	31.4	28.1	
SMART^a^	55.8	73.6	46.6	40.8	**<0.01**
**Reward**					
Recorded	23.7	30.3	24.2	16.7	0.18
What?					
*Dinner at a Restaurant*	27.7	25.0	43.8	9.1	0.66
*Alcohol/“Night Out”*	21.3	30.0	12.5	18.2	
*Special Purchase (e.g. golf driver, concert tickets)*	17.0	20.0	12.5	18.2	
*New Clothes*	10.6	5.0	12.5	18.2	
*Achieving Goals/Feeling Better*	4.3	5.0	0	9.1	
*Other*	19.1	15.0	18.8	27.3	

Data presented as %, the percentage of participants recording the specified variable. Results were combined for *Online* and *Resources* groups. *P*-value represents the comparison between months. ^a^SMART goals, reported as the percentage of participants having recording any goal. ^b^‘Junk food’ includes chips, pies, chocolate, cakes, biscuits, lollies, soft drink and ‘fast food’. ^c^Bolded *p* value is statistically significant (*p <0.05)*.

Table 3 illustrates that many men did not record strategies to engage their social support networks. Only 43% of men recorded at least one social support strategy during the first month of the program, which fell to only 21% during the third month. In addition, men did not appear to grasp the concept of social support, with 34% of the men naming themselves as their support person rather than their actual support network. Thus, only 34% of men recorded ‘quality’ social support strategies in the first month and only 11% during the third month. Overall, the most popular person men recorded in social support strategies was their partner or wife, which was expected as 86% of men in the study were either married or in a defacto relationship. The most popular type of lifestyle supports were physical activity-based such as walking, sports or attending the gymnasium. The most popular activities linked to wives/partners was walking, supporting their participation in the SHED-IT program and avoiding the purchase of ‘junk food’. In contrast, the most popular activities men reported they would undertake with their children or grandchildren were walking and bike riding. Friends were most commonly enlisted to participate in sports, attend the gymnasium and assist with reducing alcohol intake. For additional detail on the link between social support strategies and the person providing the support, see Additional file 1: Table S2.

**Table 3:** Social support strategies recorded during the SHED-IT program (n = 67)

	**Overall**	**Month 1**	**Month 2**	**Month 3**	***P*-value**^**b**^
**Strategies Recorded**					
Two	23.9	32.8	22.4	16.4	**0.04**
One	6.0	10.4	3.0	4.5	
None	70.1	56.7	74.6	79.1	
**Quality Strategies Recorded**					
Two	12.9	25.4	7.5	6.0	**<0.01**
One	8.0	9.0	10.4	4.5	
None	79.1	65.7	82.1	89.6	
**Who?**^**a**^					
Partner/Wife	36.5	47.3	27.3	25.9	**0.02**
Himself	33.9	14.5	48.5	55.6	
Child(ren)/Grandchild(ren)	15.7	20.0	15.2	7.4	
Friend(s)	6.1	9.1	3.0	3.7	
Work Colleague(s)	6.1	5.5	6.1	7.4	
Dog(s)	1.7	3.7	0	0	
**What?**^**a**^					
Walk	34.8	40.4	30.3	29.6	0.51
Sport/Gymnasium	12.5	7.7	18.2	14.8	
Avoid Purchasing Unhealthy /Fast Food	11.6	15.4	6.1	11.1	
Alcohol Intake	7.1	11.5	3.0	3.7	
Participate In/Support SHED-IT Program	5.4	3.8	6.1	7.4	
Ride Bike	2.7	3.8	3.0	0	
Other	25.9	17.3	33.3	33.3	

Data presented as %, the percentage of participants recording the specified variable. Results were combined for *Online* and *Resources* groups. Quality social support strategy defined as engaging a person other than themselves in a supportive activity. *P*-value represents the comparison between each month. ^a^Reported as the percentage of the total number of social support strategies recorded. ^b^Bolded *p* value is statistically significant (*p <0.05)*.

### Association between compliance to SCT-based tasks and study outcomes

Table 4 presents correlations between anthropometric, dietary and physical activity outcomes, and compliance with SHED-IT program SCT-based tasks. There were strong and consistent inverse correlations between program compliance and changes to body composition, whereby those who were more compliant with recording SCT-based tasks also demonstrated greater reductions in weight, waist circumference and body fat. Additionally, there was a positive correlation between compliance with monitoring dietary intake and physical activity, as well as with changes to weight, waist circumference, fat mass and dietary fat intake.

**Table 4:** Correlation between change in selected outcomes and Support Book engagement

	**Goals**	**SMART goals**	**Exercise goals**	**Nutrition goals**	**Rewards**	**Social support strategies**	**Weight record**	**Pedometer record**	**Exercise diary entries**	**Food diary entries**
Weight (kg)	−0.47***	−0.49***	−0.47***	−0.47***	−0.33**	−0.44**	−0.51***	−0.36**	−0.47***	−0.46***
Waist Circumference (cm)	−0.54***	−0.54***	−0.55***	−0.50***	−0.38**	−0.38**	−0.46***	−0.36**	−0.48***	−0.46***
Fat Mass (kg)	−0.42***	−0.46***	−0.42**	−0.42***	−0.27*	−0.32**	−0.45***	−0.39**	−0.46***	−0.45***
Energy (kJ)	0.09	0.04	0.10	0.06	0.08	0.10	−0.09	−0.16	−0.02	−0.11
Total Fat (%)	−0.04	−0.26*	−0.25*	−0.25*	−0.16	−0.10	−0.19	−0.13	−0.35**	−0.34**
SFA (%)	−0.16	−0.21	−0.17	−0.16	−0.10	−0.12	−0.17	−0.17	−0.22	−0.26*
Pedometer Steps (steps/day)	0.02	−0.03	0.00	0.03	0.16	0.16	0.16	0.20	0.10	0.20

Quality social support strategies defined as engaging a person other than themselves in a supportive activity. kJ – kilojoule; SFA – saturated fat. *p < 0.05, **p < 0.01, ***p < 0.001.

Table 5 presents results for the associations between weight loss and Support Book compliance by utilising a multiple linear regression model. Inter-correlations between the Support Book components ranged from r = 0.21 (food diary/rewards) to r = 0.68 (goals/social support) (Additional file 1: Table S3). The number of goals set (β-coefficient [95% CI] = -0.3 [-0.6, -0.1], *p* = 0.01) and the number of weight records documented (β-coefficient [95% CI] **= -**0.21 [-0.39, -0.02], *p* = 0.03) were independently associated with weight loss, whereby each additional goal set and weight measurement recorded was associated with an increased weight loss of 0.32 kg and 0.21 kg, respectively (R^2^ = 0.37, *p* <0.001). This equates to an additional 2.9 kg and 2.7 kg weight loss respectively, for those who set all their goals and monitored their weight weekly, compared to those who did not engage in these activities. Age, SES and intervention group were not significant predictors of weight change in this model (*p* > 0.05 for each).

**Table 5:** Multiple linear regression of recording various Support Book components as predictors of weight loss (kg)

**Weight change (kg)**	**Unadjusted model**		**Final model**	
			***R***^***2***^**= 0.37**	***p*** **<0.001**
**Variable**	**β-Coefficient (95% CI)**	***p*-value**	**β-Coefficient (95% CI)**	***p*-value**^**a**^
Goals (n)	−0.40 (−0.6, −0.2)	<0.001	−0.30 (−0.6, −0.1)	**0.01**
Rewards (n)	−1.10 (−1.8, −0.3)	0.011		
Social Support (n)	−0.50 (−0.9, −0.2)	0.007		
Weight Record (n)	−0.40 (−0.5, −0.3)	<0.001	−0.21 (−0.39, −0.02)	**0.03**
Food Diary Entries (n)	−0.06 (−0.09, −0.03)	<0.001		
Pedometer Record (n)	−0.30 (−0.4, −0.1)	0.001		
Age (years)	0.04 (−0.05, 0.12)	0.389	0.02 (−0.06, 0.11)	0.58
Socioeconomic Status	0.01 (−0.01, 0.03)	0.512	0.01 (−0.01, 0.03)	0.21
Intervention (*Online* vs*. Resources*)	1.60 (−0.1, 3.3)	0.072	1.30 (−0.3, 2.9)	0.11

^a^Bolded *p* value is statistically significant (*p <0.05)*.

### Participant perceptions of the SHED-IT program

A total of 38 men from the *Online* group (88.4%) and 37 men from the *Resources* group (86.0%) completed the process evaluation questionnaire at 6-months (Table 6). Overall, participants were very satisfied with the SHED-IT program. Men found the DVD and other weight loss resources engaging and enjoyable, and agreed that the program had positive effects on those around them. Although both groups indicated that the program provided them with sufficient support for weight loss, the *Online* group reported significantly stronger agreement than the *Resources* group. Most men reported reading the Weight Loss Handbook for Blokes once (26%) or 2-3 times (57%), with 91% indicating that the length of the Handbook was appropriate. Most participants (93%) felt the 25-minute DVD was an appropriate length. Sixty-five percent of men reported watching the DVD once, while 34% watched it more than once.

**Table 6:** Participant perceptions of the SHED-IT program

	**Overall**	**Online**	**Resources**	**Online vs Resources** ***p*-value**^**a**^
***The SHED-IT program***				
a) SHED-IT provided me with the support needed to lose weight	4.2 ± 0.8	4.4 ± 0.7	4.0 ± 0.9	**<0.05**
b) The SHED-IT program corrected some wrong beliefs I had about physical activity, nutrition and weight loss	4.0 ± 0.9	3.9 ± 1.0	4.1 ± 0.7	0.51
c) I now have a much better understanding of energy balance and weight loss	4.3 ± 0.6	4.3 ± 0.7	4.3 ± 0.6	0.86
d) I would recommend SHED-IT to my friends	4.6 ± 0.6	4.7 ± 0.5	4.5 ± 0.6	0.32
e) I would prefer being in a program that meets on the internet to being in one that meets in person	2.8 ± 1.2	3.0 ± 1.2	2.5 ± 1.2	0.08
f) There was too much reading to do for the SHED-IT program	2.1 ± 1.0	2.1 ± 0.9	2.1 ± 1.1	0.73
g) The Mathematics of Weight Loss was explained in a way that was easy to understand	4.3 ± 0.8	4.2 ± 0.9	4.3 ± 0.7	0.32
***SHED-IT Weight Loss DVD for Blokes***				
a) The DVD helped me understand weight loss fundamentals	4.3 ± 0.6	4.2 ± 0.6	4.3 ± 0.6	0.34
b) I found the DVD enjoyable to watch	4.2 ± 0.7	4.2 ± 0.7	4.2 ± 0.7	0.68
c) The DVD helped me to lose weight	3.8 ± 0.8	3.8 ± 0.8	3.8 ± 0.8	0.83
***Other SHED-IT Weight Loss Resources***				
a) The Weight Loss Handbook for Blokes was enjoyable to read	3.9 ± 0.8	3.9 ± 0.6	4.0 ± 0.9	0.43
d) The SHED-IT Weight Loss Support Book for Blokes was easy to understand	4.1 ± 0.8	4.1 ± 0.5	4.2 ± 0.9	0.87
e) The SHED-IT Weight Loss Support Book for Blokes helped me to lose weight	4.0 ± 0.8	3.9 ± 0.8	4.1 ± 0.8	0.31
f) The Calorie, Fat and Carbohydrate Counter book helped me to choose which foods I would eat while trying to lose weight	3.9 ± 1.1	3.6 ± 0.8	4.3 ± 1.0	**0.012**
***Effect of SHED-IT on those around me***				
a) As a result of my participation in SHED-IT, other members of my family have started to make healthier food choices	3.5 ± 1.0	3.6 ± 1.0	3.3 ± 0.9	0.09
b) As a result of my participation in SHED-IT, other members of my family have become more active	3.3 ± 1.0	3.4 ± 1.0	3.1 ± 0.9	0.12
c) As a result of my participation in SHED-IT other members of my family have lost weight	2.9 ± 0.9	3.1 ± 0.9	2.9 ± 0.8	0.28
d) As a result of my participation in SHED-IT one or more of my friends have lost weight	2.9 ± 0.9	2.9 ± 1.0	2.8 ± 1.0	0.64
e) I have had conversations with friends, co-workers and/or relatives about weight loss strategies I learned in SHED-IT	4.1 ± 0.8	4.1 ± 0.9	4.2 ± 0.8	0.60
***Calorie King Website***				
a) The Calorie King website was easy to understand	-	3.8 ± 1.0	-	-
b) Recording my daily food and exercise on the Calorie King website was time consuming	-	3.4 ± 1.0	-	-
c) The Calorie King website was a valuable tool to help me understand how to lose weight	-	4.2 ± 0.8	-	-
d) The Calorie King User Guide was useful	-	3.8 ± 0.7	-	-
e) The Calorie King User Guide was easy to follow	-	3.7 ± 0.7	-	-
***Feedback***				
a) I found the email feedback reports provided by the SHED-IT team helped me to lose weight	-	3.9 ± 0.9	-	-
b) The feedback reports were personalised enough	-	4.1 ± 0.7	-	-
c) I would have preferred fewer email feedback reports from the SHED-IT team	-	2.3 ± 0.9	-	-
d) I would have preferred more email feedback reports from the SHED-IT team	-	3.6 ± 0.8	-	-

Data reported as mean ± standard deviation of participant responses to the statements above, where: 1 = strongly disagree; 2 = disagree; 3 = neutral; 4 = agree; 5 = strongly agree. *P*-values are given for the interaction and main effects of intervention group and compliance. Program compliance is defined as returning the Support Book and recording weight ≥ 80% of the time. ^a^Bolded *p* value is statistically significant (*p <0.05)*.

In the *Online* group, the men reported that the CalorieKing™ website was useful for weight loss and was relatively easy to understand. This group also indicated that the feedback reports were helpful and assisted them in losing weight. Two-thirds of participants reported always reading their email feedback reports, while 11% read them some of the time, 14% half the time or most of the time and 6% reported to have never read them.

Most participants reported using at least five of the nine SHED-IT weight loss tips. Overall, the most commonly used weight loss tips were ‘read food labels’ (81% of participants), ‘reduce your portion sizes’ (81% of participants) and ‘reduce kJ dense snacks’ (73% of participants). *Resources* group compliers were more likely than non-compliers to use the tip ‘keep a healthy lifestyle diary’ (p = 0.02), while *Online* group compliers were more likely to use the tip ‘surf the urge’ (p = 0.01). For more information on the SHED-IT weight loss tip results, see Additional file 1: Table S4.

## Discussion

The overall aim of this paper was to conduct a comprehensive process evaluation of the previously established successful SHED-IT weight loss program for men. Our findings indicate that most men undertook SCT-based tasks including goal setting and monitoring of weight, physical activity and dietary intake. However, utilisation of social support networks and reward selection was poor. Of the SCT-based tasks, the most critical for success were goal setting and weekly weight monitoring, which were independently associated with weight loss. The *Online* and *Resources* versions of the SHED-IT program performed equally well, with no differences in weight loss, engagement in SCT-based tasks, or perception of program quality or program impact. The SHED-IT program was perceived to be supportive, enjoyable and beneficial.

A major finding of our study was the association found between various program tasks and behaviour change. While monitoring of all SCT-based tasks correlated with improved body composition, a multiple linear regression model demonstrated that goal setting and weekly monitoring of weight were the most important predictors of weight loss in this cohort of men. This model found that setting all nine goals equated to an additional weight loss of 2.9 kg and those who monitored their weight every week for the 3-month intervention had a corresponding additional weight loss of 2.7 kg compared with those who did not engage in these activities. This aligns with findings from a recent meta-regression of 122 physical activity and healthy eating behaviour change interventions, in which interventions with a self-monitoring component were significantly more effective than those without [[27]]. This finding also supports Bandura’s notion within SCT that self-regulatory behaviours are an essential component of successful behaviour change. According to Bandura (1997), self-regulatory behaviours rise to become the most important component of behaviour change when the behaviour of interest involves skills that people already know or can quickly learn (e.g. the motor skills required to perform physical activity) [[19]].

Although goal setting was an important predictor of success, it is important to note that only half of the dietary and exercise goals the men recorded were considered ‘SMART’. This is important as research has shown that setting SMART goals increases the likelihood of goal attainment whereas non-specific goals reduce self-efficacy, which has a negative impact on subsequent goal setting [[28]]. Despite explicit instructions and multiple examples, this finding suggests that men still have difficulty understanding SMART goals and will require additional support in future programs. It is plausible that men who were more ready to lose weight prior to commencing the study were more compliant with program activities, which may have created a spurious association between compliance and weight loss. However, a recent systematic review of goal setting and behaviour change in adults supported our finding, by demonstrating that goal setting leads to changes in dietary and exercise behaviours [[28]]. Similarly, regular self-weighing enables self-evaluation of progress and further reinforces desired behaviours, thus contributing to weight loss [[28]]. In addition, a number of recent papers have demonstrated that self-regulation is the most powerful construct within SCT to explain and predict physical activity behaviour [[29]-[33]], dietary behaviour [[30],[34]] and weight loss [[35]] across a variety of populations. Therefore, the current research supports our observation, suggesting an important role for both regular self-weighing and goal setting in promoting weight loss in men.

While, in general, men completed the designated SCT-based tasks, most did not document rewards for achieving their goals. Furthermore, the proportion of men engaging in this activity reduced by half between the first and third month of the intervention. The reason for this lack of reward documentation during the SHED-IT program is unclear, but may be because the participants did not find the activity helpful for weight loss, chose to keep the rewards to themselves, lost motivation over time, or had an initial lack of ideas for good rewards. Alternatively, some men may have viewed weight loss as the reward without needing to motivate themselves with additional extrinsic rewards. Interestingly, when men did set rewards, the majority involved going out for dinner, drinking alcohol, or having a night out. This supports previous research demonstrating that men value weight loss programs that are not overly restrictive [[36]], and that allow them to socialise and enjoy energy-dense luxuries on occasion [[10]].

Overall, most men did not engage with the social support task in the Support Book. Less than 25% of men recorded strategies to engage social networks, and one-third of these were deemed to be poor quality because they recorded themselves as their support person rather than other people. Although the reason for the poor uptake of this activity is not known, it is possible that its importance was not sufficiently emphasised, that the activity was not well understood, or that the men’s previous exposure to this style of documentation or thinking was limited. In addition, sociological literature on men’s health shows that men prefer to engage in lifestyle programs that they can complete independently [[37]], and thus the men may not have valued the idea of asking for help or support. Despite this, social support has been found to increase both intervention compliance and weight loss [[38],[39]]. As such, identifying strategies that improve compliance with this activity may be warranted. It is also important to note that social support is not the only socio-structural factor outlined in Bandura’s SCT. However, targeting and operationalising this construct remains a difficult task as it represents a multitude of factors above and beyond social support networks [[40]]. These include ethnic group membership, education and intelligence, socio-economic status, the built environment and access to local facilities [[41]]. To improve study outcomes, future studies should explore novel ways to operationalise this construct above engaging social support networks alone.

Compliance or adherence to program tasks has been acknowledged as a major limitation of weight loss interventions, with the Internet as an engagement medium being identified as a particular risk. Indeed, attrition rates have been reported to exceed 40% for internet-based weight loss research [[42]]. Despite this, the current study had an attrition rate of only 20% for both intervention groups, which is similar to the average attrition rate of 21% for weight loss interventions in general. This suggests that, despite having no face-to-face contact, SHED-IT is as effective as other weight loss program formats in men. In addition, this is supported by our findings that overall weight loss, program engagement, perception of program quality, and program impact were similar for both the online self-monitoring and paper-based self-monitoring versions of SHED-IT.

A significant strength of the SHED-IT weight loss community program was its theoretical structure. Most weight loss interventions are not theory-driven, or use theory only as a loose framework, which is known to weaken the intervention effects [[43]]. As noted previously, the SHED-IT program was informed by Bandura’s SCT and was constructed to explicitly target the key behaviour change mediators specified in the theory. A systematic review of studies using the Internet as a medium for delivering health behaviour change programs demonstrated that, while only 20% of interventions used or mentioned theory as an intervention technique, a greater use of theory improved effect sizes, particularly those extensively incorporating theory into their program [[43]]. This review also demonstrated that goal setting, self-monitoring, social support and reward provision all have a significant, positive influence on behaviour change [[43]]. While all SCT-based tasks correlated with improved body composition in the current study, goal setting and weight monitoring were the strongest predictors of weight loss. Therefore, the combination of SCT-tasks used in this study improved weight outcomes in men.

Overall, men found the program to be beneficial and to provide sufficient support for their weight loss endeavours. Of particular interest, the men reported that the DVD was enjoyable to watch, improved their understanding of how to lose weight and approximately one-third of men watched the DVD more than once. These are important findings, given that the DVD was introduced to improve the scalability of the intervention by replacing the face-to-face information session of the pilot study [[13]]. Of note, both groups reported similar engagement with the program and both groups indicated that they would recommend SHED-IT to a friend, suggesting that the online self-monitoring and paper-based self-monitoring versions of SHED-IT were equally effective in engaging men. Interestingly, compliance with exercise monitoring was greater for the *Online* group compared to the *Resources* group. Although this may have been a chance finding due to the number of comparisons tested, it may also have been a function of task-difficulty. The online exercise diaries utilised established exercise databases and automatically calculated kilojoules burned during exercise, which eliminated the need to perform time-consuming calculations [[44]]. Therefore, the findings of the current process evaluation support the use of the Internet as an effective medium for weight loss intervention in men and suggest further examination is warranted.

A limitation of this study is some participants did not return their Support Book and the extent to which these were completed cannot be determined. However, the overall retention of participants in the trial was strong [[9]]. In addition, this study cannot confirm whether completion of the Support Book resulted in greater weight loss, because the Support Books were collected immediately prior to the assessment and not monitored throughout the intervention period. This could be investigated in future trials. This study contained several strengths including recruitment of a community sample of men, comprehensive tailoring of intervention materials to appeal to men and use of an extensively validated behaviour change theory. Aside from assessments, the SHED-IT programs involved no face-to-face contact and were considerably lower in intervention intensity than previous male-only weight loss studies [[9]]. The current analyses allowed for a comprehensive process evaluation, which provides valuable information to inform the development of novel obesity treatment strategies that engage men.

Currently, there is a dearth of research regarding how to engage men in weight loss programs and how to create effective programs that are tailored to their interests. Although we have previously established the efficacy [[13]] and effectiveness [[17]] of the SHED-IT program, this was the first study to determine which program components were most strongly associated with success. In addition to informing future refinements of the program, the current findings may help to encourage and inform other weight loss interventions for men, which are urgently required [[5],[9]]. In summary, based on the findings of the current process evaluation, we make the following recommendations for the development of such interventions:

1. Face-to-face information sessions or tailored intervention components may not be required for men to feel sufficiently supported in weight loss programs. Despite being considerably lower in intensity than other programs, men felt that the *Online* and *Resources*-only modalities provided them with sufficient support to lose weight.

2. The quality of strategies (e.g. gender tailoring selection of behaviour change techniques,) and information in the resources may be more important for men than a particular treatment modality. Both the *Online* and *Resources* groups were equally satisfied with the program and would have recommended the program to their friends.

3. Goal setting and weekly weight tracking were found to be the key program components associated with success for men.

4. Men may require additional information, examples or strategies to be able to successfully set S.M.A.R.T. goals, particularly for dietary and physical activity behaviours.

5. Future studies should pay attention to men’s initial weight loss expectations, particularly in the context of setting goals. Initially, approximately 25% of men who set a weight-related goal were aiming to lose more than the recommended ½ to 1 kg per week and 33% of men did not achieve their first target.

6. Despite being key behaviour change strategies outlined in Bandura’s SCT [[18],[40]], setting social support strategies and rewards for success were not associated with improved treatment outcomes for men. However, it is important to note that men experienced considerable conceptual confusion with the social support task, with approximately 50% of men listing themselves as their own social support.

7. For men, physical activity goals were predominantly related to accessible lifestyle activities such as walking/ increasing step counts. Future studies with men should include a strong focus on these activities, which do not require specialised knowledge or skills.

8. When trying to improve eating habits, the most common goal for men was to reduce junk food intake, particularly in the first month. Given the large proportion of men’s energy intake that comes from non-essential foods [[45]], this is an important finding and these goals could be encouraged in future programs

## Conclusion

Given the increasing prevalence of overweight and obesity in men and the lack of targeted weight loss interventions conducted in this population, the design and evaluation of effective and acceptable programs is imperative. There is a need for appealing and widely accessible programs for men and this process evaluation provides strong direction for the design of such trials. This study demonstrates that Bandura’s SCT provides a useful framework to design weight loss interventions for men, and future studies should include a strong focus on self-regulatory behaviours such as self-monitoring and goal setting to enhance behaviour changes and improve treatment effects. Further investigation into the value that men place on social support during weight loss is warranted, with most men in the current study not complying with this aspect of the program. Overall, SHED-IT provides an effective medium for delivering a weight loss program tailored to men’s preferences.

## Abbreviations

BMI: Body Mass Index

RCT: Randomised Controlled Trial

SCT: Social Cognitive Theory

SES: Socio-economic status

SHED-IT: Self-help, Exercise and Diet using Information Technology

SMART: Specific, measurable, attractive, realistic, time-targeted

## Competing interests

The authors declare that they have no competing interests.

## Authors’ contributions

PJM, RC, CEC, RCP were responsible for identifying the research questions, design of the study, obtaining ethics approval, the acquisition of funding and overseeing study implementation. MDY contributed to development of intervention materials, recruiting participants and study implementation. HAS completed the statistical analysis for the current study. All authors were responsible for the drafting of this manuscript and have read and approved the final version.

## Additional file

## Supplementary Material

Additional file 1: Table S1. Baseline characteristics of participants completing the intervention phase of the SHED-IT program. **Table S2.** Link between social support activities and the person providing support. **Table S3.** Inter-correlations among completion rates for the support book components. **Table S4.** The proportion of participants who reported using the nine weight loss tips to assist with weight loss. Click here for file
