# Supplementary material for: Associations between program outcomes and adherence to Social Cognitive Theory tasks: process evaluation of the SHED-IT community weight loss trial for men
Source: Int J Behav Nutr Phys Act. 2014 Jul 11;11:89. doi: 10.1186/s12966-014-0089-9 (PMC4105870; doi:10.1186/s12966-014-0089-9)
Supplement: Additional file 1: Table S1. — Baseline characteristics of participants completing the intervention phase of the SHED-IT program. Table S2. Link between social support activities and the person providing support. Table S3. Inter-correlations among completion rates for the support book components. Table S4. The proportion of participants who reported using the nine weight loss tips to assist with weight loss. [file s12966-014-0089-9-S1.doc]

| **Table S1.** Baseline characteristics of participants completing the intervention phase of the SHED-IT program. | | | | |
| --- | --- | --- | --- | --- |
|  | **Overall (n=67)** | **Online (n=32)** | **Resources (n=35)** | ***P*-value** |
| Age (years) | 47.3±10.9 | 48.2±10.0 | 49.8±9.1 | 0.48 |
| Weight (kg) | 103.2±13.5 | 102.6±15.0 | 101.2±12.7 | 0.68 |
| BMI (kg/m^2^) | 32.6±3.3 | 32.3±3.4 | 32.0±3.5 | 0.73 |
| Waist Circumference (cm) | 113.1±9.4 | 112.8±10.5 | 111.9±9.7 | 0.72 |
| SES, n (%) |  |  |  |  |
| *1-2 (lowest)* | 7 (6.5) | 2 (6.3) | 2 (5.7) | **0.03** |
| *3-4* | 15 (14.0) | 8 (25.0) | 1 (2.9) |
| *5-6* | 41 (38.8) | 10 (31.3) | 13 (37.1) |
| *7-8* | 33 (30.8) | 7 (21.9) | 17 (48.6) |
| *9-10 (highest)* | 11 (10.3) | 5 (15.6) | 2 (5.7) |
| Energy Intake (kJ/day) | 11547±3496 | 11026±3211 | 10266±4071 | 0.33 |
| Physical Activity (steps/day) | 6950±2962 | 7625±3255 | 5336±2725 | 0.17 |
| Data are presented as mean±SD or n (%) the number and percentage of subjects with the specified variable. BMI – body mass index; SES – socioeconomic status; kJ – kilojoules. | | | | |

| **Table S2.** Link between social support activities and the person providing support | | | | | | |
| --- | --- | --- | --- | --- | --- | --- |
|  | **Partner/Wife** | **Himself** | **Child(ren)/**  **Grandchild(ren)** | **Friend(s)** | **Work Colleague(s)** | **Dog(s)** |
| Walk | 37.2 (16) | 27.5 (11) | 52.9 (9) | 12.5 (1) | 57.1 (4) | 100.0 (2) |
| Sport/Gymnasium | 4.7 (2) | 20.0 (8) | 5.9 (1) | 37.5 (3) | 0 (0) | 0 (0) |
| Avoid Purchasing Unhealthy /Fast Food | 18.6 (8) | 10.0 (4) | 5.9 (1) | 12.5 (1) | 0 (0) | 0 (0) |
| Alcohol Intake | 9.3 (4) | 5.0 (2) | 0 (0) | 25.0 (2) | 0 (0) | 0 (0) |
| Participate In/Support SHED-IT Program | 14.0 (6) | 0 (0) | 0 (0) | 0 (0) | 14.3 (1) | 0 (0) |
| Ride Bike | 0 (0) | 0 (0) | 11.8 (2) | 12.5 (1) | 0 (0) | 0 (0) |
| Other | 16.3 (7) | 37.5 (15) | 23.5 (4) | 0 (0) | 28.6 (2) | 0 (0) |
| Data are presented as % (N) of activities reported for each person to provide social support. | | | | | | |

| **Table S3**. Inter-correlations among completion rates for the support book components. | | | | | |
| --- | --- | --- | --- | --- | --- |
|  | 1. | 2. | 3. | 4. | 5. |
| 1. Goals (n) | 1.00 |  |  |  |  |
| 2. Rewards (n) | 0.66** | 1.00 |  |  |  |
| 3. Social Support (n) | 0.68** | 0.67** | 1.00 |  |  |
| 4. Weight Record (n) | 0.53** | 0.31* | 0.49** | 1.00 |  |
| 5. Food Diary Entries (n) | 0.27* | 0.21 | 0.31* | 0.58** | 1.00 |
| 6. Pedometer Record (n) | 0.43** | 0.36** | 0.45** | 0.50** | 0.51** |
| * *p*<0.05, ** *p*<0.01 | | | | | |

| **Table S4.** The proportion of participants who reported using the nine weight loss tips to assist with weight loss | | | | | | | | | | |
| --- | --- | --- | --- | --- | --- | --- | --- | --- | --- | --- |
|  |  |  | **Online** | | |  |  | **Resources** | | |
|  | **Overall** |  | **Compliers**  **(n=19)** | **Non-Compliers (n=19)** | ***P*-value** |  |  | **Compliers**  **(n=16)** | **Non-Compliers (n=21)** | ***P*-value** |
| Read Food Labels | 81.3 |  | 78.9 | 77.8 | 0.93 |  |  | 93.8 | 76.2 | 0.15 |
| Reduce your portion sizes | 81.3 |  | 84.2 | 66.7 | 0.21 |  |  | 93.8 | 81.0 | 0.26 |
| Reduce kJ dense snacks | 73.3 |  | 78.9 | 66.7 | 0.40 |  |  | 87.5 | 61.9 | 0.08 |
| Every step counts | 64.0 |  | 68.4 | 61.1 | 0.64 |  |  | 68.8 | 57.1 | 0.47 |
| Don’t drink you kilojoules | 54.7 |  | 47.4 | 55.6 | 0.62 |  |  | 68.8 | 47.6 | 0.20 |
| Keep a healthy lifestyle diary | 46.7 |  | 47.4 | 50.0 | 0.87 |  |  | 68.8 | 28.6 | **0.02** |
| Reduce your sitting time | 37.3 |  | 26.3 | 38.9 | 0.41 |  |  | 43.8 | 42.9 | 0.96 |
| Be prepared | 34.7 |  | 31.6 | 22.2 | 0.52 |  |  | 50.0 | 38.1 | 0.47 |
| Surf the urge | 32.0 |  | 52.6 | 11.1 | **<0.01** |  |  | 43.8 | 23.8 | 0.20 |
| Data presented as %, the percentage of participants reporting having used the specified weight loss tip. *P*-value compares compliers vs. non-compliers for both the Online and Resources groups. | | | | | | | | | | |
